# Vitamin D and diabetic foot ulcer: a systematic review and meta-analysis

**DOI:** 10.1038/s41387-019-0078-9

**Published:** 2019-03-11

**Authors:** Jiezhi Dai, Chaoyin Jiang, Hua Chen, Yimin Chai

**Affiliations:** Department of Orthopedic Surgery, Shanghai Jiao Tong University Affiliated Sixth People’s Hospital, Shanghai, China

## Abstract

We aimed to evaluate the association between vitamin D deficiency and diabetic foot ulcer (DFU) in patients with diabetes. Pubmed, EMBASE, BIOSIS, the Cochrane Library, and Web of Knowledge, last updated in July 2018, were searched. We assessed eligible studies for the association between vitamin D deficiency and DFU in diabetic patients. The mean difference (MD) or the odds ratio (OR) was calculated for continuous or dichotomous data respectively. Data were analyzed by using the Cochrane Collaboration’s RevMan 5.0 software. Seven studies that involved 1115 patients were included in this study. There were significantly reduced vitamin D levels in DFU (MD −13.47 nmol/L, 95%CI −16.84 to −10.10; *P*  =  0.34, *I*^2^ = 12%). Severe vitamin D deficiency was significantly associated with an increased risk of DFU (OR 3.22, 95%CI 2.42−4.28; *P*  = 0.64, *I*^2^ = 0%). This is the first meta-analysis demonstrating the association between serum vitamin D levels and DFU. Severe vitamin D deficiency is significantly associated with an increased risk of DFU.

## Introduction

The diabetic foot ulcer (DFU) is a severe complication in patients with diabetes mellitus (DM)^1^. Patients with DFU have a higher mortality compared with diabetic patients without foot ulcer. Daousi et al.^2^ reported the mortality rate of DFU is about twice that of nonulcerated diabetic patients. However, there is a paucity of information that explores possible contributory factors on DFU.

In recent years, there is an increasing interest in the beneficial role of vitamin D in DM. Some researches have reported its effect on T-cell-mediated immunity, pancreatic insulin secretion and action as well as cell growth and healing^3^. Yakob et al.^4^ found the low levels of vitamin D may be related to the development of diabetic foot infections. Findings from in vitro studies reported that vitamin D could restore the production of antimicrobial peptides in primary cell from DFU and improve the in vitro wound-healing assays^5^. In rats, the topical application of vitamin D accelerated wound healing in a dose-dependent manner^6^. Another study found that calcitriol could promote endothelial and keratinocyte cell migration in a DFU model^7^. Thus, findings from previous studies showed the possible beneficial effects of vitamin D on wound healing in DFU. However, there were no conclusive results available. In this study, we conducted a review and meta-analysis of cohort studies to assess the relationship between serum vitamin D levels with DFU.

## Materials and methods

### Study selection

Pubmed, EMBASE, BIOSIS, the Cochrane Library, and Web of Knowledge were searched for studies published before July 2018. The following combinations of search terms were used: (vitamin D, or 25-hydroxyvitamin D) AND (diabetic foot, DFU, or diabetic foot infection). There were no limitations on language and year of publication. After titles and abstracts review, studies deemed potentially eligible for inclusion were further evaluated by full text reading. Reference lists of the retrieved articles were also reviewed. Two reviewers (J.D. and C.J.) independently conducted the literature search, and disagreements were resolved through discussion with the third authors.

### Inclusion and exclusion criteria

All eligible studies must meet the following inclusion criteria: (a) nested case-control study or cohorts study; (b) analyzing the relationship between serum vitamin D levels and DFU; (c) the outcomes were serum vitamin D level or rate of severe vitamin D deficiency; (d) adequate data for extracting or calculating. In addition, the exclusion criteria were as follows: (a) case reports, reviews, editorials and (b) animal studies.

### Data extraction

We extracted all data with a standardized data-collection protocol. For each study, two investigators independently extracted information on: information on study design, last name of the first author, publication year, country, number of patients, gender, age, serum vitamin D level, rate of severe vitamin D deficiency, and duration of follow-up.

As primary analysis, we assessed mean vitamin D levels in patients with DFU and in control group. As secondary analysis, we evaluated the prevalence of severe vitamin D deficiency in patients with DFU. It is widely accepted that normal 25-hydroxyvitamin D3 supplies a minimal serum value of 20 ng/mL (50 nmol/L)^8^. Serum level less than 20 ng/mL (50 nmol/L) was classified as “vitamin D deficiency” and that less than 10 ng/mL (25 nmol/L) was defined as “severe vitamin D deficiency”.

### Statistical analysis

Study analyses were performed by the Cochrane Collaboration’s RevMan 5.0 software. Statistical analysis of continuous variables was carried out by using mean difference (MD) with 95% confidence intervals (CIs), and odds ratio (OR) with 95% CIs was used for dichotomous data. Serum vitamin D level is expressed in ng/mL or nmol/L (1 ng/mL = 2.496 nmol/L). Between-study heterogeneity was tested by Q statistics and *P* < 0.05 indicated that there was heterogeneity present between studies. *I*^2^ statistic method was also used and *I*^2^ > 50% indicated high heterogeneity.

A sensitivity analysis was performed by repeating the analysis after sequential exclusion of one study at a time.

The Newcastle-Ottawa Scale (NOS) was used to assess the quality of the included studies^9^. The scoring system includes three major areas: participants’ selection, study comparability, and outcomes. Study with 6 or more score indicates higher quality.

Potential publication bias was evaluated by funnel plots, the Begg’s rank correlation test^10^, and the Egger’s regression test^11^. Data were analyzed using STATA 10.0 software. A *P* value less than 0.05 was considered statistically significant.

## Results

### Study selection and features

**Fig. 1:**
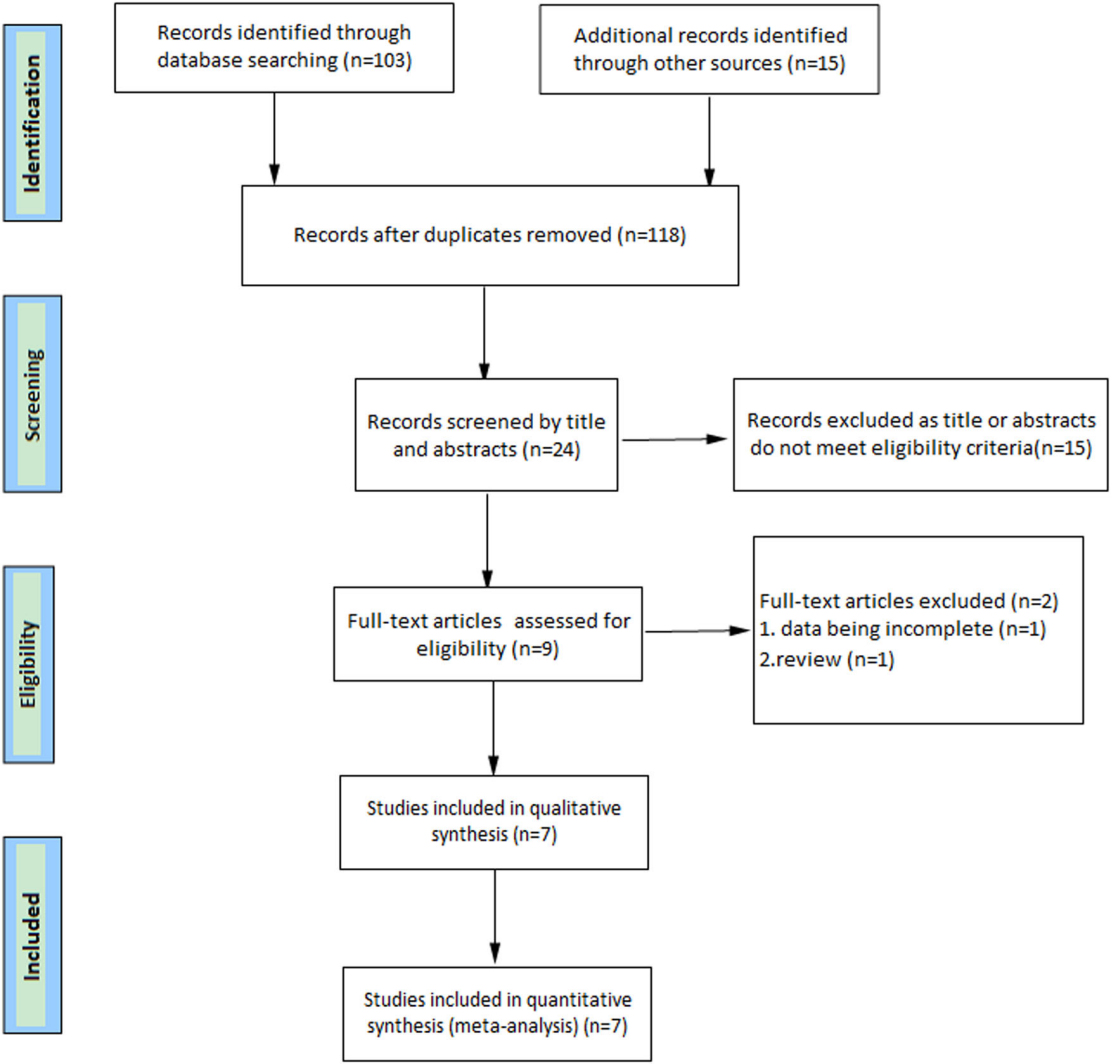
Flow chart of study selection in the systematic review

A total of 143 primary studies were identified from online databases prior to July 2018. According to the inclusion and exclusion criteria, seven articles^12–18^ were included (Fig. 1). Six studies^12,13,15–18^ were published in English, and one^14^ was published in Russian. Both men and women were included in each study. The number of cases diagnosed in the primary studies ranged from 40 to 289. Both men and women were included in each study. All identified studies followed an observational design, with five retrospective cohorts and two prospective cohorts. The characteristics and quality evaluation of included studies are shown in Table 1.

**Table 1:** Characteristics of included studies

	Country	Study design	Age (mean)		Patients (No.)		Vitamin D (nmol/L)		Vitamin D < 10 ng/mL		NOS	Vitamin D assay
			DFU	DM	DFU	DM	DFU	DM	DFU	DM		
Afarideh et al.^16^	Iran	Prospective	59	54.5	30	30	41.93 ± 45.48	39.94 ± 26.07	NR	NR	7	Elisa
Feldkamp et al.^17^	Germany	Retrospective	70.2	69.4	104	103	29.45 ± 28.2	47.42 ± 35.94	57	32	8	RIA
Gupta and Singh^13^	India	Retrospective	54.56	49.62	50	50	35.57 ± 21.12	53.11 ± 27.41	NR	NR	6	RIA
Ignatovich et al.^14^	Russia	Prospective	NR	NR	22	18	27.75 ± 10.25	43.75 ± 9.25	NR	NR	7	Elisa
Kota et al.^12^	India	Retrospective	54.3	52.5	100	100	40.18 ± 39.94	49.42 ± 35.19	48	26	6	RIA
Tiwari et al.^15^	India	Retrospective	53.6	51	125	164	40.25 ± 38.35	50.75 ± 33	57	28	6	RIA
Tiwari et al.^18^	India	Retrospective	53.6	51.9	112	107	40.2 ± 39.2	49.4 ± 33.1	54	22	6	RIA

*RIA* radioimmunoassay, *NR* not reported, *DFU* diabetic foot ulcer, *DM* diabetes mellitus, *NOS* the Newcastle−Ottawa Scale

### Vitamin D levels in patients with DFU and DM groups

**Fig. 2:**
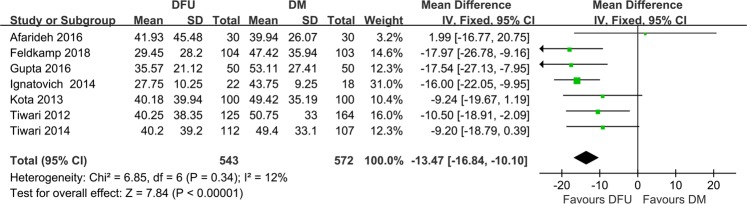
Meta-analysis of vitamin D levels in patients with DFU and DM group

**Fig. 3:**
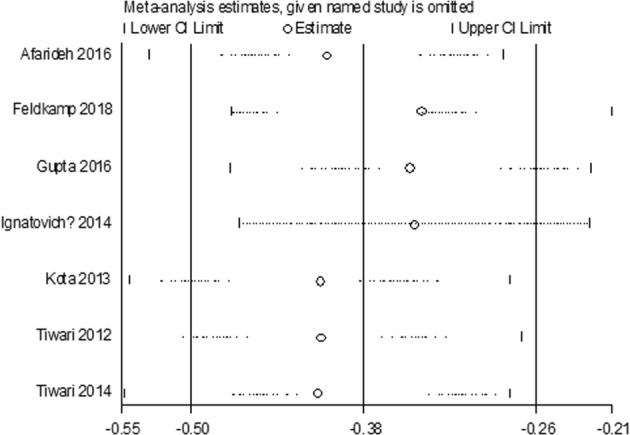
Sensitivity analysis of the meta-analysis

Seven studies reported significantly lower serum vitamin D levels in 543 patients with DFU than in the 572 DM group (MD −13.47 nmol/L, 95%CI −16.84 to −10.10, *P* = 0.34) (Fig. 2). There was no between-study heterogeneity among these studies (*I*^2^ = 12%, *P* = 0.34), and sensitivity analyses showed that the summary results were not significantly influenced by any single study (Fig. 3).

### DFU associated with severe vitamin D deficiency

**Fig. 4:**
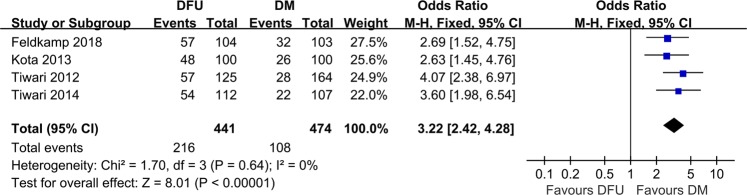
Meta-analysis of DFU associated with severe vitamin D deficiency

Four studies reported data on severe vitamin D deficiency. Severe vitamin D deficiency was reported in 216 (48.98%) patients with DFU and 108 (22.78%) in the DM group. No substantial heterogeneity was observed (*I*^2^ = 0%). Based on the forest plot, severe vitamin D deficiency (25-OHD < 10 ng/mL) was significantly associated with the risk of DFU (OR 3.22, 95%CI 2.42−4.28; *P* = 0.64, *I*^2^ = 0%) (Fig. 4).

**Fig. 5:**
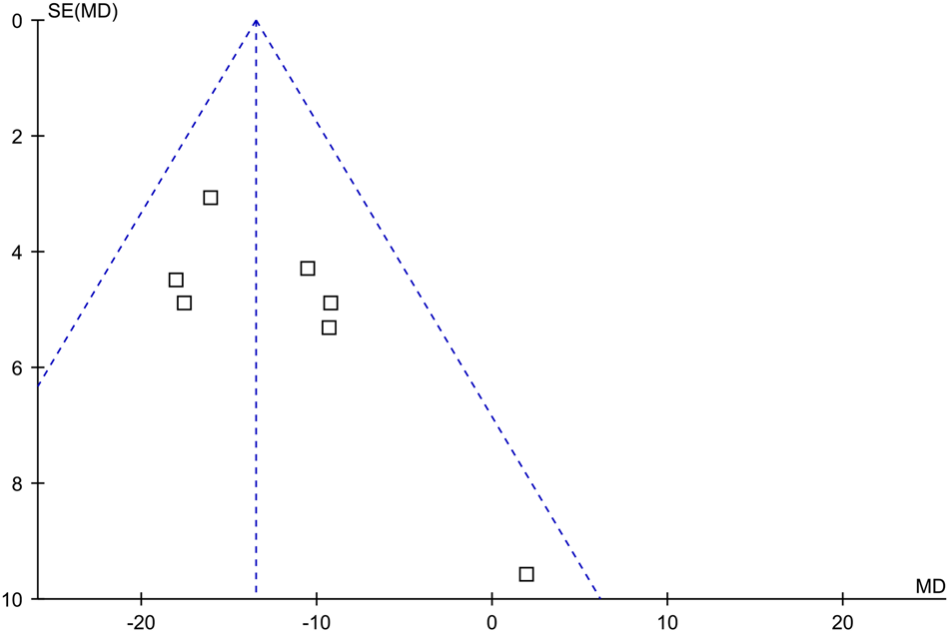
Publication bias analysis of the meta-analysis

The funnel plot in the meta-analysis showed no evidence of publication bias (Fig. 5). It was also indicated by Egger’s test (*P* = 0.217) and Begg’s test (*P* = 0.764).

## Discussion

In this study, we conducted a meta-analysis of seven articles with 1115 patients, which provided a comprehensive evaluation of the association between vitamin D and diabetic foot. Meta-analysis reported a significantly lower vitamin D levels in patients with DFU compared with the DM group. Severe vitamin D deficiency was definitely associated with an increased risk of DFU.

Vitamin D has been suggested to play an important role in many chronic diseases, such as diabetes^19^. Low serum vitamin D levels are associated with insulin resistance, impaired β-cell function, and the development of DM^20,21^. There is also an ongoing interest in the association between lower level of vitamin D and diabetic complications. Zubair et al.^22^ found that lower 25(OH)D played a significant role in the pathogenesis of foot ulcer. Multiple mechanisms were involved in this process. Hyperglycemia in diabetic patients disturbed the normal process of cytokine production, which in turn impaired wound healing^23^. Vitamin D supplementation was reported to improve gylcemic control. Sugden et al.^24^ reported the effect of vitamin D in the improvement of endothelial function. A single high dose of oral vitamin D could substantially improve the brachial artery flow-mediated vasodilatation by 2.3%. Moreover, vitamin D has been suggested as an immune stimulant^25^. Urashima et al.^26^ suggested that vitamin D supplementation can reduce the risk of influenza A in schoolchildren. Van et al.^27^ found vitamin D stimulated the phagocytosis and killed the intestine bacteria by macrophages. Vitamin D also inhibited the secretion of the T helper type 1 cytokines IFN-γ and IL-2 while stimulated the production of Th2 cytokines, which may promote wound healing^28^. However, the mechanism of the relationship between serum vitamin D levels and DFU is still unclear.

This study was the first meta-analysis aiming to estimate the relationship between vitamin D levels and DFU. It showed a significant reduction in vitamin D levels related to DFU with a mean difference of −13.47 nmol/L (95%CI −16.84 to −10.10). Our findings consistently supported the association between vitamin D levels and DFU, and also performed separate analyses for severe vitamin D deficiency. In the independent analyses, we found an increased incidence of DFU for declining levels of vitamin D. Patients with severe vitamin D deficiency had a higher prevalence (48.98 vs. 22.78%) in diabetic foot patients, as compared with those in the DM group. Therefore, the identification of the association of DFU with vitamin D can give us some implications to develop new therapy for DFU. Vitamin D supplementation may be a valid therapeutic option for diabetes with foot ulcer and vitamin D deficiency. It is also supported by the only RCT that evaluated effects of vitamin D supplementation for DFU patients^29^. More future studies are required to verify the effect of vitamin D supplementation in the prevention or treatment of DFU.

Potential limitations of this study should be discussed. Firstly, we included only seven studies and the sample size of individual studies was small. It showed that the quantity of researched objects was insufficient. Secondly, two prospective and five retrospective cohort studies were included. Existence of bias was unavoidable in this research. In future, more well-designed trials with large number of participants are urgently required.

## Conclusion

In conclusion, this is the first meta-analysis demonstrating the association between serum vitamin D levels and DFU. Severe vitamin D deficiency is significantly associated with an increased risk of DFU. More specifically designed studies are needed in the future.
